# Intraoperative arthroscopic classification tool for posterolateral elbow instability

**DOI:** 10.1016/j.jseint.2023.02.016

**Published:** 2023-04-13

**Authors:** Elisabeth A. Wörner, Mustafa Kaynak, Roger van Riet, Bertram The, Denise Eygendaal

**Affiliations:** a Department of Orthopaedic Surgery, Erasmus MC, Rotterdam, The Netherlands; b Department of Orthopaedic Surgery, Amphia Hospital, Breda, The Netherlands; c Department of Orthopaedic Surgery, AZ Monica hospital, Deurne, Belgium

**Keywords:** Posterolateral elbow instability, Arthroscopy, Classification, Instability, Elbow, PLRI, Ulnohumeral joint

## Abstract

**Background:**

Introducing and implementing an arthroscopic classification tool for posterolateral elbow instability.

**Methods:**

Thirty arthroscopies were performed on 30 patients, and all recordings were collected, blinded, and labeled. Three orthopedic surgeons reviewed and scored all 30 recordings three times with a period of at least seven days in between to analyze the intraobserver and interobserver reliability. The classification consisted of five different grades.

**Results:**

Indications for elbow arthroscopy included impingement (n = 7), osteochondritis dissecans (n = 5), pain (n = 7), osteoarthritis (n = 6), and other (n = 5). The kappa value for intrarater reliability was 0.71, indicating good reliability, while the kappa value for inter-rater reliability was 0.38 indicating fair reliability.

**Conclusion:**

This new classification is a tool for an arthroscopic assessment of PLRI and can be used as a standardized grading system for further research and communication between orthopedic surgeons. We demonstrated good intrarater reliability (k = 0.71) with fair inter-rater reliability (k = 0.38). However, further research is necessary to study the clinical significance.

Posterolateral elbow instability (PLRI) was first described in 1991 by O'Driscoll et al as a rotatory subluxation of the ulnohumeral joint with a secondary dislocation of the radiohumeral joint. A loss of function of the lateral collateral ligament complex and surrounding tissues can cause PLRI.^12^ It is a typical pattern of elbow instability characterized by a history of elbow trauma or iatrogenic damage to the lateral-sided structures of the joint. The most common symptoms are recurrent dislocations or sublocations, painful giving way, clicking, snapping, or locking of the elbow.^1^^,^^9^, ^10^, ^11^, ^12^ Physical examination is the primary diagnostic tool for PLRI. In addition, various diagnostic imaging methods are available to diagnose PLRI, such as computed tomography scan and plain radiographs. Examination under anesthesia, with or without fluoroscopy or even arthroscopy, can be used as a diagnostic tool for preplanning a formal ligamentous reconstruction.^4^^,^^9^ Arthroscopy might reveal posterior displacement of the radial head, laxity in the lateral ligament complex, and widening of the lateral joint space, together with sagging of the annular ligament.

In the case of gross elbow instability, the “drive-through sign” is positive. In this case, the scope is easily driven from the posterolateral portal across the ulnohumeral articulation into the medial aspect of the joint. In addition, the “arthroscopic pivot shift sign” is evaluated and is usually clearly positive in these cases. Decision-making in these elbows is relatively straightforward for arthroscopic repair or open reconstruction.^17^^,^^18^ However, clinical and radiographic signs of instability are subtle in many cases, even during arthroscopic evaluation. An arthroscopic classification tool could help grade the severity of instability objectively. In addition, a classification tool may help better understand normal and pathologic lateral-sided ligamentous behavior under direct view and provide orthopedic surgeons with a means to communicate using a standardized grading system. We have developed an arthroscopic classification system for grading the severity of posterolateral elbow instability. This study reports on the development of this classification and its standardized use in practice. The interobserver and intraobserver reliability was assessed and reported.

## Materials and methods

### Patients

Between August 2015 and October 2015, 30 arthroscopies were performed on 30 patients. All patient characteristics are shown in Table I. Patients are men and women with a mean age of 44.2 and 33.9 years, with various indications for elbow arthroscopy. To determine our population size, we used the method described by Walter and Donner.^5^ We determined a P0 >0.6 would be acceptable with α = 0.05 and β = 0.20. The number of observations is three, and we calculated the optimal number of subjects to be 27. To minimize the effect of data loss, we chose our sample size to consist of 30 recorded elbow arthroscopies in which the classification was performed.

**Table I:** Study parameters.

Parameter	
Sex	
Male, n	N = 22
Female, n	N = 8
Mean age	
Male, y	44.2
Female, y	33.9
Elbow side	
Right, n	N = 19
Left, n	N = 11

### Study design

All recordings of the arthroscopies were collected, blinded, and labeled with a number. Three orthopedic surgeons viewed and scored all 30 recordings three times, with at least seven days in between. All three orthopedic surgeons are board-certified and fellowship-trained elbow surgeons with more than five years of clinical experience. The surgeries were carried out by one surgeon, who provided the data. The order in which the surgery recordings were reviewed was randomized to minimize recognition and memorization of the recordings.

### Surgical technique

The patient is positioned in lateral decubitus with the shoulder in 90 degrees of forward flexion and the elbow in 90 degrees of flexion with the forearm in neutral rotation. After carefully inspecting and marking the anatomy, including the course of the lateral ulnar collateral ligament, the arm is disinfected and draped. A 4 mm, 30 degrees angle scope was used. The arthroscopy proceeds in a standard fashion, completing the necessary procedures in the anterior and posterior compartments as needed. To obtain an excellent and standardized view of the ulnohumeral joint, the scope is introduced into the soft spot through the lateral gutter via the posterolateral tunnel. A soft spot portal is then created to introduce a 4 mm obturator. Care is taken only to incise the skin while perforation of the capsule is completed, using a small blunt instrument to protect the ligamentous complex on the lateral side. If necessary, a 4 mm shaver blade can first be used to establish a clear view, again being careful not to damage the lateral ulnar collateral ligament injury. Next, the arthroscopy view was positioned through the posterolateral portal. The 4 mm (blunt) obturator is then introduced into the soft spot portal and advanced until touching the ulnohumeral joint at the bare area. It is then—without using force—advanced from lateral to medial into the ulnohumeral joint.

### Classification

**Figure 1:**
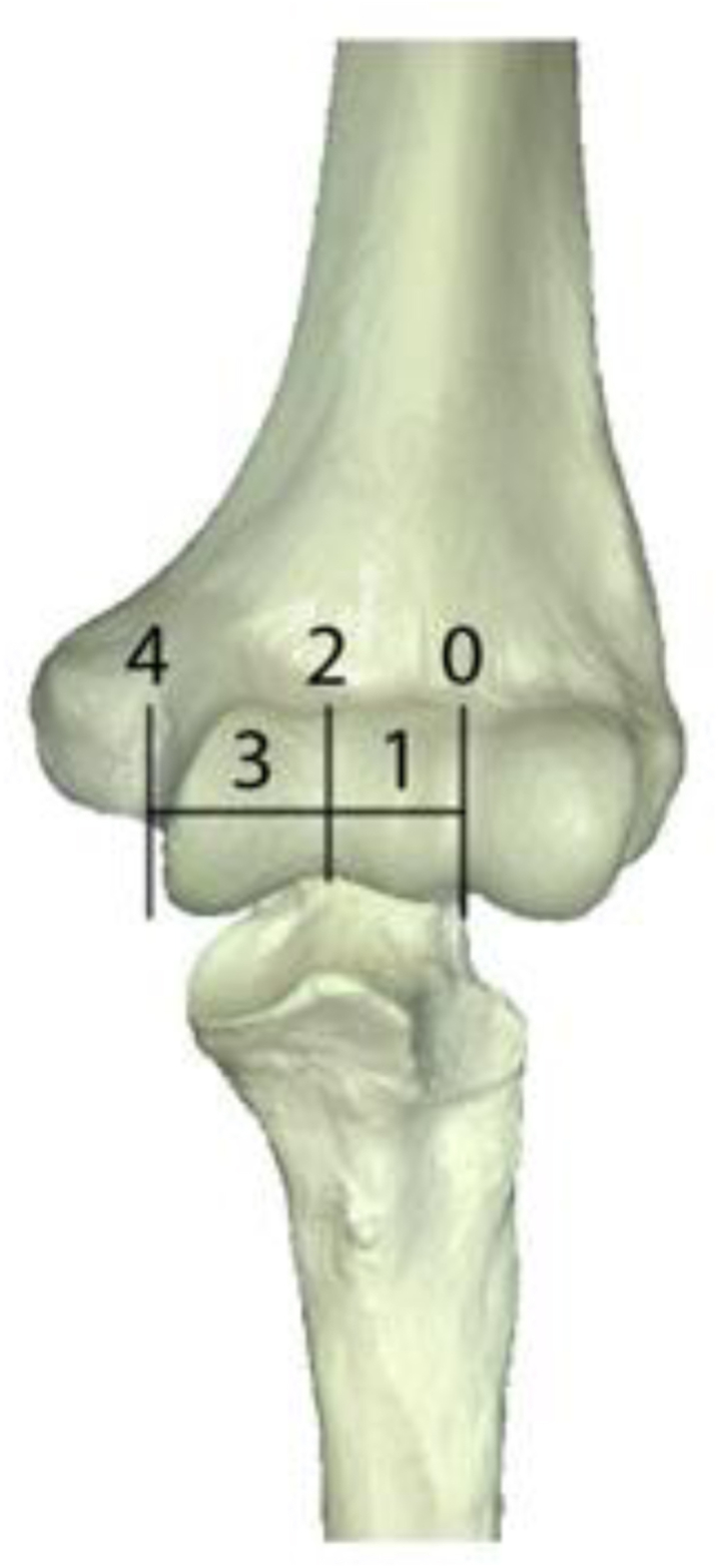
Anatomic location of the grading.

**Figure 2:**
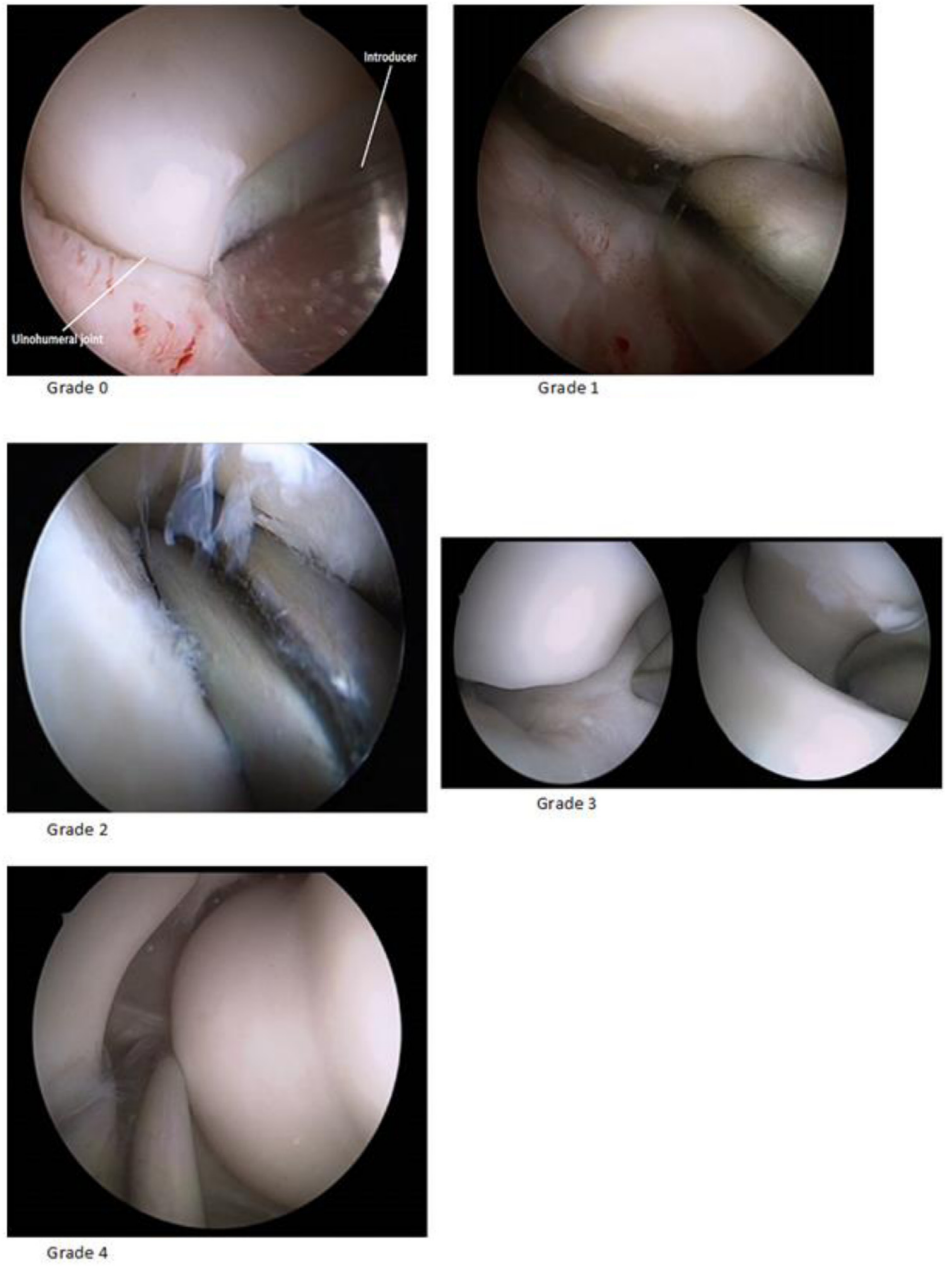
Arthroscopic view of the different grades.

The classification is divided into five grades, 0 to 4 (Figs. 1 and 2):

Grade 0 – The obturator cannot be inserted in the ulnohumeral joint.
Grade 1 – Introduction of the obturator is possible but unable to reach the trochlear groove (<50%).
Grade 2 – The obturator can be advanced up to the trochlear groove (50%).
Grade 3 – The obturator advances beyond the trochlear groove (>50%), but no drive-through sign is present.
Grade 4 – “Drive-through sign.”

The complementary Video 1 provides a clear view of the findings concerning posterolateral instability of the elbow (grade 0-4).

### Data analysis and statistics

All score sheets were gathered, and the data were analyzed using SPSS (IBM Corp., Armonk, NY, USA) and Excel (Microsoft Corp., Redmond, WA, USA). We used Cohen's kappa to determine the intraobserver agreement and Gwet's and Fleis' kappa to determine the interobserver agreement. The results were compared using the criteria of Landis and Koch: kappa values can vary from −1 to +1, where agreement values are labeled as follows: <0.20 poor; 0.21-0.40 fair; 0.41-0.60 moderate; 0.61-0.80 good; and 0.81-1.00 very good.

## Results

Details on the indication for surgery and distribution of instability grades are provided in Tables II and III. The most frequent indication for elbow arthroscopy was post-traumatic pain (n = 6) and impingement (n = 7), followed by primary osteoarthritis (n = 6), osteochondritis dissecans (n = 6), and other (n = 5). Fourteen patients had experienced a previous elbow trauma in which the arthroscopy was performed. Nine of these patients (surgical n = 3, nonsurgical n = 6) had previously received treatment. Perioperatively, grade 1 (n = 10) was the most encountered elbow instability score, while grade 4 (n = 2) was the least frequent. The kappa value for intrarater reliability was 0.71, indicating good reliability, while the kappa value for inter-rater reliability was 0.38, indicating fair reliability.

**Table II:** Parameters included elbows.

Parameter	n
Indication for elbow arthroscopy	
Impingement	7
Post-traumatic pain	6
Primary osteoarthritis	6
OCD	6
Other	5
Previous elbow trauma	
Elbow luxation	4
Radial head fracture	6
Olecranon fracture	1
Coronoid fracture + elbow luxation	1
Unspecified elbow fracture	1
Unspecified trauma	1
No fracture	16
Previous elbow treatment	
Surgery	3
Nonsurgical treatment	6
No treatment	20
Unknown	1

*OCD*, osteochondritis dissecans.

**Table III:** Perioperative grading.

Initial grade perioperative	n
Grade 0	5
Grade 1	10
Grade 2	8
Grade 3	5
Grade 4	2

## Discussion

The objective of this study was to introduce a new arthroscopic classification tool for PLRI and to test the reliability and reproducibility of the classification. The kappa value for intrarater reliability was 0.71, indicating good reliability, while the kappa value for inter-rater reliability was 0.38, indicating fair reliability. No arthroscopic classification is currently available to rate posterolateral instability; therefore, we could not test this tool on an existing classification tool. Although the intraobserver reliability is good, the tool needs to be studied further for clinical use because the interobserver reliability is fair. It should be noted that other frequently used classification tools also show fair to moderate interobserver agreement, such as the AO/Neer classifications tool for proximal and humeral shaft fractures or Weber/AO/Lauge-Hansen for malleolar fractures.^8^^,^^13^^,^^19^

The primary diagnostic tool for PLRI is a physical examination. Several tests have been developed: the lateral pivot-shift and apprehension tests, the posterolateral rotatory drawer test, the chair, and push-up signs, and the tabletop relocation test.^2^^,^^7^^,^^10^^,^^12^^,^^14^

Plain radiographs and computed tomography scans can be used in acute and chronic cases to assess the integrity of stabilizing osseous structures. Stress radiographs can be made to confirm instability, but the sensitivity is low.^10^ The use of ultrasound and magnetic resonance imaging is controversial. They can show the extent of the soft tissue damage and scar tissue in the lateral structures. Still, they can also show ligamentous integrity and be interpreted as normal, even in clear clinical PLRI.^6^ Additionally, examination under anesthesia, with or without fluoroscopy or even arthroscopy, can be used as a diagnostic tool and for the preplanning of a formal ligamentous reconstruction.^4^^,^^9^ Arthroscopy is a relatively minimal invasive useful diagnostic tool to evaluate the elbow joint with additional possible pathology of the surrounding structures (ligaments, joint capsule, arthritis, and instability). This method provides a classification method for PLRI and anticipates surgical action for any other elbow pathology if necessary. The degree of posterolateral instability was graded accordingly. Still, this classification tool has yet to determine the clinical consequences of the different types of grades except for grade 4 (drive-through sign). Future studies must provide more insight into which of these grades are regarding clinical consequences (and indication for surgery).

As previously stated, no other published studies describe the arthroscopic classification of posterolateral elbow instability, limiting our ability to compare our results. However, other studies report the reliability of arthroscopic grading in the musculoskeletal system other than the elbow. Thus, it is possible to compare the interobserver and intraobserver reliability agreements to identify the clinical significance of our results. The intraobserver (0.42-0.66) and interobserver (0.43-0.49) agreements in the arthroscopic classification of knee osteoarthritis indicated an inaccurate observer reliability.^3^ Another study regarding the arthroscopic grading of cartilage lesions concerning the knee concluded poor interobserver reliability, with no data on intraobserver reliability.^16^ The inter-rater variability regarding arthroscopic grading in patients with anterior shoulder instability was very good concerning the anterior labrum, supraspinatus tendon, and detecting Hill-Sachs lesions. However, it was poor concerning the glenoid and anterior inferior glenohumeral ligament.^15^ Unfortunately, this study also reported no data concerning intraobserver variability.

The strength of this study is blinding and randomization in which the procedure has been taken to minimize bias. We included 30 patients, and three orthopedic surgeons scored the arthroscopy recordings, which was sufficient for statistical power. All three orthopedic surgeons were experienced and specialized in pathological conditions of the elbow and surrounding structures ensuring qualitative reviews as high as possible. A limitation of this study is the heterogenous population group in which we performed the arthroscopies varying from degenerative cases to post-traumatic fractures. We refrained from analyzing subgroups since there were not enough patients to analyze the results of these subgroups statistically; therefore, the study design was lacking. Future studies should include more patients to analyze subgroups.

Regarding the surgical procedure, the arthroscopy was positioned in 90° of flexion, while PLRI is clinically most apparent in extension. The degree of instability may eventually differ in flexion (arthroscopy position) compared to extension (in which PLRI appears). Finally, this was an assessment of reliability and reproducibility based on clinicians watching one surgeon doing the arthroscopy.

Elbow instability remains a topic of interest. Various classification tools are designed and practiced to diagnose or preclude posterolateral instability.^10^ However, none of these classifications include grading with the assistance of arthroscopy. As previously noted, PLRI may be challenging to access because of the subtle clinical and radiographic signs of instability. We attempted to introduce a new classification tool, which may provide orthopedic surgeons with more insight into the degree of PLRI independent of clinical and radiographic characteristics. For this classification tool, future studies should perform this classification tool in a homogenous selected population compared to clinical examinations of PLRI.

## Conclusion

This study was the first attempt to introduce a new arthroscopic classification tool concerning posterolateral rotatory instability of the elbow to provide orthopedic surgeons with a means to communicate using a standardized grading system. It will aid in a better understanding of normal and pathologic lateral-sided ligamentous behavior under direct view. Our results demonstrate good inter-rater reliability (k = 0.71) and fair intrarater reliability (k = 0.38) while using the drive-through sign in an unselected population group.

## Disclaimers

Funding: No sources of funding were used to assist in the preparation of this article.

Conflicts of interest: The authors, their immediate families, and any research foundation with which they are affiliated have not received any financial payments or other benefits from any commercial entity related to the subject of this article.

The editor making the decision to accept this paper for publication had no conflicts of interest related to the decision. Further, peer review of this paper was handled independently of any author of this paper.
